# Association Between Telehealth and Missed Appointments Among Patients Experiencing Behavioral Health Challenges

**DOI:** 10.1001/jamanetworkopen.2023.24252

**Published:** 2023-07-19

**Authors:** Dependra Bhatta, Monteic A. Sizer, Binod Acharya

**Affiliations:** 1 Northeast Delta Human Services Authority, Monroe, Louisiana; 2 Urban Health Collaborative, Drexel University, Philadelphia, Pennsylvania

## Abstract

This cohort study compares missed appointments between patients receiving telehealth and in-person care in behavioral health clinics in rural settings that cater to patients with relatively low income.

## Introduction

Missed appointments, or no-shows, can affect patients’ health due to delays in receiving timely care and result in inefficient use of health care resources. Telehealth has generally been considered effective and is widely implemented to treat behavioral health conditions. Although knowledge about no-shows is critical to inform health care institutions for effective management of resources and better health outcomes,^1^ a limited body of literature has compared no-shows between telehealth and in-person care in behavioral health clinics in rural settings that cater to patients with relatively low income.

## Methods

This cohort study is a retrospective analysis of electronic health records of patients with behavioral health conditions who scheduled appointments in outpatient clinics in rural Louisiana from May 1, 2022, to January 31, 2023. The description of clinics and community context is provided elsewhere^2^; additional details are in the eMethods in Supplement 1. This research followed the STROBE reporting guideline. Propensity scores were estimated by adjusting for gender, age, primary target group (mental health, substance use, or co-occurring), health insurance (Medicaid, Medicare, or other), residence type (private or other), marital status, employment status, and source of referral (self or other). Kernel matching was performed between the treated group (telehealth users) and the control group (in-person users) such that each treated observation was matched against a weighted composite of the control pool, in which control observations are weighted by their distance in propensity score from the treated observation.^3^ Logistic regression analysis was performed to calculate the average treatment effect (ATE) of telehealth (vs in-person care) on no-shows in the matched sample. The Louisiana Department of Health institutional review board considered this study exempt from review and waived the consent requirement because the data were deidentified. Statistical tests were performed with Stata, version 17 (StataCorp LP). All *P* values were from 2-sided tests and results were deemed statistically significant at *P* < .05.

## Results

Our analysis included 9715 appointments (3318 in-person and 6397 telehealth) involving 1421 patients 18 years or older (929 [65%] were telehealth users; 804 [57%], women; 478 [34%], aged 31-45 years; 267 [19%], without private residence; 801 [56%], never married; and 1075 [76%], unemployed) (Table 1). The no-show rate was 13% for in-person appointments (436 of 3318) and 17% for telehealth appointments (1108 of 6397). The standardized mean differences in the matched sample were less than 10% for all variables, demonstrating a good covariate balance. Comparison of no-shows between patients using telehealth and in-person care after matching indicated that patients in the telehealth cohort had statistically significantly higher odds of no-shows than patients in the in-person cohort (odds ratio of ATE for the treated, 1.42 [95% CI, 1.09-1.84]) (Table 2).

**Table 1.** Covariate Balance Before and After Matching^a^

Patient characteristic	Original sample			Kernel-matched sample		
	Telehealth (n = 929)	In person (n = 492)	SMD, %	Telehealth (n = 921)	In person (n = 492)	SMD, %
Gender, No. (%)						
Male	381 (41)	236 (48)	−14	387 (42)	212 (43)	−2
Female	548 (59)	256 (52)	14	534 (58)	280 (57)	2
Age group, y, No. (%)						
18-30	223 (24)	157 (32)	−19	239 (26)	138 (28)	−4
31-45	325 (35)	153 (31)	9	313 (34)	162 (33)	1
46-60	260 (28)	138 (28)	1	267 (29)	133 (27)	5
>60	121 (13)	44 (9)	12	101 (11)	59 (12)	−3
Primary target group, No. (%)						
Mental health	687 (74)	413 (84)	−25	728 (79)	394 (80)	−4
Substance use	93 (10)	15 (3)	30	64 (7)	34 (7)	1
Co-occurring	149 (16)	64 (13)	8	129 (14)	64 (13)	4
Health insurance, No. (%)						
Medicaid	622 (67)	354 (72)	−10	626 (68)	344 (70)	−4
Medicare	158 (17)	69 (14)	8	147 (16)	84 (17)	−1
Other	149 (16)	69 (14)	5	147 (16)	64 (13)	7
Residence type, No. (%)						
Private or independent	790 (85)	364 (74)	28	746 (81)	403 (82)	−3
Otherwise	139 (15)	128 (26)	−28	175 (19)	89 (18)	3
Marital status, No. (%)						
Never married	511 (55)	290 (59)	−8	516 (56)	276 (56)	−2
Otherwise	418 (45)	202 (41)	8	405 (44)	216 (44)	2
Employment status, No. (%)						
Employed	223 (24)	123 (25)	−2	221 (24)	113 (23)	2
Unemployed	706 (76)	369 (75)	2	700 (76)	379 (77)	−2
Source of referral, No. (%)						
Self	381 (41)	231 (47)	−12	405 (44)	212 (43)	2
Otherwise	548 (59)	261 (53)	12	516 (56)	280 (57)	−2

Abbreviation: SMD, standardized mean difference.

^a^ Propensity score kernel matching at bandwidth 0.005 reduced the SMD in the matched sample.

**Table 2.** Association Between Telehealth Use and No-Show Status in Matched Data

Status (no-show)	Odds ratio (95% CI)	*P* value
Average treatment effect	1.29 (1.02-1.62)	.03^a^
Average treatment effect for the treated	1.42 (1.09-1.84)	.009^a^

^a^ Statistically significant at *P* < .05.

## Discussion

These results suggest that, although telehealth was widely implemented after the beginning of the COVID-19 pandemic to limit the spread of the virus, it may have unintentionally prompted patients experiencing behavioral health challenges to miss their scheduled appointments at a rate higher than it would have been for in-person care. Although the reasons behind no-shows can be multifaceted, difficulty in establishing a therapeutic patient-clinician relationship, loss of nonverbal cues and psychological support, and unfamiliarity with the use of technology may discourage patients from attending telehealth sessions for mental health.^4^ The main limitation of this study is that we focused on behavioral health clinics serving rural populations; therefore, the findings may not be generalizable to community contexts with different levels of the digital divide and socioeconomic status.^5^ This study analyzed no-shows in rural Louisiana, where 19% of the patients did not have stable housing and many lacked private residential space, internet connectivity, a sufficient cellular data plan, and stable income—all constituting some forms of unmet social needs—which may present barriers to telehealth and can be associated with increased no-shows.^6^ Reducing no-shows should incorporate strategies to address unmet social needs along with programs such as sending reminders, offering flexible scheduling, and providing incentives.

## Notes

Supplement 1. **eMethods.** Supplemental Methods
eReferences

Supplement 2.
Data Sharing Statement
