# Supplementary material for: Association Between Telehealth and Missed Appointments Among Patients Experiencing Behavioral Health Challenges
Source: JAMA Netw Open. 2023 Jul 19;6(7):e2324252. doi: 10.1001/jamanetworkopen.2023.24252 (PMC10357338; doi:10.1001/jamanetworkopen.2023.24252)
Supplement: Supplement 1. — eMethods. Supplemental Methods eReferences [file jamanetwopen-e2324252-s001.pdf]

Supplementary Online Content

Bhatta D, Sizer MA, Acharya B. Association between telehealth and missed appointments among patients experiencing behavioral health challenges. *JAMA Netw Open.* 2023;6(7):e2324252. doi:10.1001/jamanetworkopen.2023.24252

eMethods. Supplemental Methods

eReferences

This supplementary material has been provided by the authors to give readers additional information about their work.

© 2023 Bhatta D et al. *JAMA Network Open.*

eMethods. Supplemental Methods

Study population

This is a retrospective cohort analysis of electronic health records of patients with behavioral health conditions who made at least one appointment in the six outpatient clinics of Northeast Delta Human Services Authority (NEDHSA) between May 1, 2022, through January 31, 2023. NEDHSA is a local government entity that provides behavioral health services, mainly to the residents of Louisiana's twelve northeastern rural parishes. NEDHSA widely implemented telehealth services in March 2020 following the Substance Abuse and Mental Health Services Administration (SAMSHA) and Louisiana State's recommendations. During the study window of nine months, the NEDHSA outpatient clinics also provided in-person care from 8 am to 4:30 pm during business days and it was the patient's choice to decide whether to opt for telehealth or an in-person visit. Qualified medical professionals, psychiatrists, and advanced practitioner psychiatric nurses diagnose the behavioral health condition of patients considering DSM-5 diagnostic criteria[1]. Appointments were categorized as shows, no-shows, or canceled appointments. Canceled appointments refer to the instances where patients inform NEDHSA at least 24 hours before their scheduled sessions that they are unable to attend. These canceled appointments were excluded from this study. Appointments were defined as shows if patients were seen by providers or therapists. No-show visits refer to the situation where patients fail to attend their scheduled appointments without any prior cancellation or rescheduling. Patients received appointment reminders a day before the scheduled day through individual phone calls. When a patient utilized both telehealth and in-person services, different approaches are described in the literature to classify the patient as an in-person or telehealth user, depending on the visit modality the patient opted for [2,3]. For this study, we classified the patient as either telehealth or

© 2023 Bhatta D et al. *JAMA Network Open.*

in-person based on the category that accounted for more than 50% of their total visits. Telehealth included both telephone and video conferencing, although the majority of telehealth services were provided via telephone.

Statistical Analysis

Our overarching goal in this study is to estimate the association between service delivery modality (telehealth vs. in-person visits) and the no-show status of patients using the methods of propensity score matching. As such, our *treated group* refers to telehealth users, while the in-person users form a group of potential *controls*. To address the problem of potential selection bias due to non-random treatment assignment, as is common in observational studies, we use the propensity score method, which makes it easier to isolate the treatment effect by balancing covariates between individuals in the treatment group and the control group [4]. Propensity scores were estimated using the logistic regression model, with the treatment (telehealth vs. in-person visit) as the outcome variable and potential confounders as explanatory variables. These covariates include gender, age, primary target group (indicates one of the three primary diagnosis group of the patient at the time of admission, *viz.* patients experiencing mental health, patients with substance use problems, or patients with co-occurring conditions of both mental health and substance use), type of health insurance (Medicaid, Medicare, or other), residence type (private independent or other), marital status (never married or other), employment status (employed or unemployed), and source of referrals (self or other). Variables were selected on the basis of prior knowledge and theoretical considerations that help balance the treated and control cohorts and increase the precision of treatment effect [4,5]

Once the propensity scores were estimated, we performed kernel matching whereby each treated observation is matched against a weighted composite of the control pool, where these

© 2023 Bhatta D et al. *JAMA Network Open.*

control observations are weighted by their distance in propensity score from the treated observation within a bandwidth [6,7]. This method has added advantage over traditional propensity matching methods (e.g., 1:1 match) when the sample size of potential control is relatively modest [8]. We explored various bandwidth values and found that 0.005 yielded the optimal covariate balance and common support. A balance diagnostic test was performed to verify a similar distribution of scores among the treated (telehealth) and control (in-person) groups. Finally, we performed the logistic regression to calculate the average treatment effect of telehealth (vs. in-person) on no-shows. Statistical analyses were done in STATA version 17.0 with the *kmatch ps* command. This study was considered exempt from the Institutional Review Board, Louisiana Department of Health.

© 2023 Bhatta D et al. *JAMA Network Open.*

eReferences

1. American Psychiatric Association. *Diagnostic and Statistical Manual of Mental Disorders, Fifth Edition, Text Revision (DSM-5-TR®)*. 2022.
2. Gao C, Osmundson S, Malin BA, Chen Y. Telehealth Use in the COVID-19 Pandemic: A Retrospective Study of Prenatal Care. *Stud Health Technol Inform*. 2022;290:503.
3. Anindya K, Lee JT, McPake B, Wilopo SA, Millett C, Carvalho N. Impact of Indonesia's national health insurance scheme on inequality in access to maternal health services: A propensity score matched analysis. *Journal of global health*. 2020;10(1)
4. Garrido MM, Kelley AS, Paris J, et al. Methods for constructing and assessing propensity scores. *Health Serv Res*. 2014;49(5):1701-1720.
5. Sizer MA, Bhatta D, Acharya B, Paudel KP. Determinants of Telehealth Service Use among Mental Health Patients: A Case of Rural Louisiana. *Int J Environ Res Public Health*. 2022;19(11):6930.
6. Heckman JJ, Ichimura H, Todd PE. Matching as an econometric evaluation estimator: Evidence from evaluating a job training programme. *The review of economic studies*. 1997;64(4):605-654.
7. Jann B. KMATCH: Stata module for multivariate-distance and propensity-score matching. 2017;
8. Berg GD. An application of kernel-based versus one-to-one propensity score matching for a nonexperimental causal study: example from a disease management program evaluation. *Applied Economics Letters*. 2011;18(5):439-447.

© 2023 Bhatta D et al. *JAMA Network Open.*
