# Supplementary material for: Association Between Telehealth and Missed Appointments Among Patients Experiencing Behavioral Health Challenges
Source: JAMA Netw Open. 2023 Jul 19;6(7):e2324252. doi: 10.1001/jamanetworkopen.2023.24252 (PMC10357338; doi:10.1001/jamanetworkopen.2023.24252)
Supplement: Supplement 2. — Data Sharing Statement [file jamanetwopen-e2324252-s002.pdf]

Data Sharing Statement

Bhatta. Association Between Telehealth and Missed Appointments Among Patients Experiencing Behavioral Health Challenges. *JAMA Netw Open*. Published July 19, 2023. doi:10.1001/jamanetworkopen.2023.24252

Data
Data available: No

Additional Information
Explanation for why data not available: Agency policy does not allow to share the patient level data.
